# EYE MOVEMENTS REVEAL AGE DIFFERENCES IN HOW AROUSAL MODULATES MECHANISMS OF ATTENTIONAL CONTROL

**DOI:** 10.1093/geroni/igae098.2652

**Published:** 2024-12-31

**Authors:** Andy Kim, Kristine Nguyen, Mara Mather

**Affiliations:** University of Southern California Leonard Davis School of Gerontology, Los Angeles, California, United States; University of Southern California Leonard Davis School of Gerontology, Los Angeles, California, United States; University of Southern California Leonard Davis School of Gerontology, Los Angeles, California, United States

## Abstract

The arousal-biased competition theory posits that inducing arousal increases attentional priority of salient stimuli while reducing priority of non-salient stimuli. However, older adults are hypothesized to exhibit dysfunction in the locus coeruleus-noradrenaline system that mediates both global arousal and attentional control. Unlike in young adults, older adults primarily do not exhibit shifts in attentional priority under increased arousal. However, the mechanism of how arousal biases attention in older adults is conflicting. We investigated whether the threat of unpredictable shock exhibits age differences in modulating multiple mechanisms of attentional control by observing eye movements. Participants completed two oculomotor search tasks in which the salient distractor is reactively disengaged (singleton search) or proactively suppressed (feature search). During singleton search, young adults made more first saccades toward the salient distractor when under the threat of shock. However, older adults did not exhibit changes in attentional priority for any stimuli under increased arousal. Our findings reveal older adults do not exhibit elevated priority for salient stimuli because arousal does not lead to shifts in priority. Furthermore, we observed that arousal did not affect the speed of attention processing in both age groups and that proactive suppression of the distractor persisted despite elevated priority. Lastly, we demonstrated that threat of shock modulated the locus coeruleus-noradrenaline system as seen with pupillometry and that evoked pupil responses are correlated with oculomotor function. Using a systems neuroscience approach, our findings demonstrate age differences in how the locus coeruleus-noradrenaline system interacts with neural networks of attention and oculomotor function.

